# SIRT7 regulates hepatocellular carcinoma response to therapy by altering the p53-dependent cell death pathway

**DOI:** 10.1186/s13046-019-1246-4

**Published:** 2019-06-13

**Authors:** Jie Zhao, Ann Wozniak, Abby Adams, Josiah Cox, Anusha Vittal, Jordan Voss, Brian Bridges, Steven A. Weinman, Zhuan Li

**Affiliations:** 1 Department of Internal Medicine, University of Kansas Medical Center, Mailstop 1018, Kansas City, KS 66160 USA; 2 Liver Center, University of Kansas Medical Center, Kansas City, KS 66160 USA

**Keywords:** Histone deacetylase, HCC, TACE resistance, NOXA, Deacetylation

## Abstract

**Background:**

Optimal therapeutic strategies for hepatocellular carcinoma (HCC) patients are still challenging due to the high recurrence rate after surgical resection and chemotherapy resistance. Growing evidence shows that genetic and epigenetic alterations are involved in HCC progression and resistance to therapy, however the molecular mechanisms underlying resistance to therapy have not been fully understood.

**Methods:**

Expression of SIRT7 in 17 paired paraffin-embedded HCC tissues and adjacent nontumoral liver tissues was examined by immunohistochemistry and Western blot. The mRNA expression of SIRT7 in 20 paired frozen HCC tissues and adjacent nontumoral liver tissues was analyzed by quantitative RT-PCR. The biologic consequences of overexpression and knockdown of SIRT7 in HCC therapy sensitivity were studied in vitro and in vivo. Interaction between SIRT7 and p53 were studied in HCC cell lines.

**Results:**

SIRT7 expression was frequently upregulated in clinical HCC samples, and its expression was highly associated with TACE-resistance and poor survival *(P* = *0.008.)* Depletion of SIRT7 from multiple liver cancer cell lines significantly increased doxorubicin toxicity while overexpression of SIRT7 largely abolished doxorubicin induced apoptosis. At the molecular level, we observed that SIRT7 interacts with and induces deacetylation of p53 at lysines 320 and 373. Deacetylated p53 showed significantly less affinity for the NOXA promoter and its transcription. In mouse xenografts, SIRT7 suppression increased doxorubicin induced p53 activation, inhibited tumor growth and induced apoptosis.

**Conclusion:**

The newly identified SIRT7-p53-NOXA axis partially illustrates the molecular mechanism of HCC resistance to therapy and represents a novel potential therapeutic target for HCC treatment.

**Electronic supplementary material:**

The online version of this article (10.1186/s13046-019-1246-4) contains supplementary material, which is available to authorized users.

## Background

Hepatocellular carcinoma (HCC) is the most common (> 85%) liver malignancy in adults and ranks fifth in all cancer incidence and second in cancer motility worldwide [1]. Over the past 20 years, the HCC incidence rate has increased steadily in the United States. In addition, HCC incidence among young persons has increased strongly [2]. Despite considerable improvement in diagnosis and treatment for intrahepatic lesions, treatment of HCC still faces challenges. Surgical resection, orthotopic liver transplantation and microwave ablation are currently considered to be the best curative options for treatment of HCC but this requires an early stage of disease diagnosis and satisfactory liver function [3]. Unfortunately, due to the lack of signs and symptoms at early stages, HCC is often diagnosed at relatively advanced stage when it is no longer amenable to curative surgery or ablation. For treatment of intermediate stages of HCC, transarterial chemoembolization (TACE) with doxorubicin or anthracycline-based drugs providing loco-regional therapy are the main treatment options [4]. For treatment of advanced stages of HCC, systemic chemotherapy with the kinase inhibitors sorafenib, regorafenib and lenvatinib [5] and the anti PD-1 agents nivolumab and pembrolizumab are the only approved therapies but patient responses to these therapies can be poor [4]. Frequently HCC is refractory to chemotherapy as tumors acquire drug resistance and present with intrahepatic recurrences and distant metastasis [6]. Therefore, research focusing on improving understanding of the molecular mechanisms that underlying HCC response to therapy will have prime importance for HCC treatment.

SIRT7 is a family member of the silent information regulator (Sir2) proteins that are described as NAD^+^-dependent class III histone deacetylases (HDAC3). Compared with other family members like SIRT1 and SIRT6, the enzymatic activity and functions of SIRT7 are poorly understood. SIRT7 is the only Sir2 family member that is predominantly localized in the nucleus where it regulates RNA polymerase I transcription by acting as an H3K18 deacetylase [7]. Besides H3K18, SIRT7 has also been reported to target several non-histone proteins, including p53 [8], GABP-β [9], FOXO3 [10] and U3-55 k [11] for deacetylation, and has been implicated in multiple cellular functions including hepatic lipid metabolism, mitochondrial homeostasis and adipogenesis. A number of recent publications have also implicated SIRT7 in various aspects of cancer biology [12–14]. Barber et al. [12] reported that acetylated H3K18 is a target of SIRT7, and H3K18 deacetylation by SIRT7 is important for maintaining the fundamental properties of the cancer cell phenotype. SIRT7 expression correlates with cell growth, being high in metabolically active cells and low or even absent in non-proliferating cells [15, 16]. Knockdown of SIRT7 influences cell cycle control and causes an increased proportion of cancer cells that remain in the G1/S phase [7, 12, 13]. In epithelial prostate carcinomas, high SIRT7 levels are associated with aggressive cancer phenotypes, metastatic disease and poor prognosis [14]. In HCC, SIRT7 expression was upregulated in a large cohort of HCC patents and SIRT7 expression is regulated by the tumor suppressors miR-125a and miR-125b [13]. However, the functional role of SIRT7 in human HCC remain largely unknown.

In the present study, we investigated the role of SIRT7 in therapy sensitivity of HCC and our findings revealed that high SIRT7 levels are associated with therapy resistance in human HCC. Our data also suggest that SIRT7 suppresses p53 activity and prevents the p53-dependent apoptosis pathway in response to doxorubicin. Reducing SIRT7 activity significantly increased doxorubicin toxicity both in vitro and in vivo. The current findings present a novel mechanism that controls p53 activation and reveals SIRT7 as a pivotal regulatory factor in determining therapy sensitivity in human HCC. Therapeutic strategies that inhibit SIRT7 may offer novel options for the treatment of HCC.

## Materials and methods

### Cell culture, plasmids and transfection

Huh7.5 cells (provided by Dr. Charles Rice, Rockefeller University, New York, NY, USA), HepG2 cells (provided by Dr. Tiangang Li, University of Kansas Medical Center, Kansas, KS), Hep3B cells (provided by Dr. Stanley Lemon, University of North Carolina, Chapel Hill, NC) and HeLa cells (provided by Dr. Robert A. Davey, University of Texas-Medical Branch, Galveston, TX) were maintained in Dulbecco’s modified Eagle’s medium (Invitrogen, Grand Island, NY) containing 10% fetal bovine serum (FBS), 50 U/ml penicillin and 50 mg/ml streptomycin. Flag-SIRT7, Flag-SIRT7-H187Y, Flag-p53, PG13-luc (wt p53 binding sites) plasmids were respectively provided by Drs. Eric Verdin, Katrin Chua, Thomas Roberts and Bert Vogelstein via Addgene (Cambridge, MA). To generate HA-SIRT7, Flag-SIRT7 was used as template and Flag-tag was reconstituted with HA tag by using the Q5 Site-Directed Mutagenesis Kit from New England BioLabs (Ipswich, MA). p53 KR mutations were generated by Q5 Site-Directed Mutagenesis Kit. Cells were transfected in serum-free medium (Opti-MEM, Invitrogen) by using X-tremeGENE™ HP DNA Transfection Reagent (Roche, Indianapolis, IN) as previously described [17]. siRNA targeting SIRT7 (SMARTpool: ON-TARGET plus human SIRT7 siRNA) was purchased from GE Dharmacon (Lafayette, CO) and shRNA targeting SIRT7 (MISSION® shRNA: TRCN0000359594, TRCN0000359663) was purchased from Sigma-Aldrich (St. Louis, MO).

### Animal model

Male NSG mice (4 weeks of age) were purchased form The Jackson Laboratory (Bar Harbor, ME). Mice were housed in a temperature-controlled, pathogen-free environment with 12-h light-dark cycles. All animal handing procedures were approved by the Institutional Animal Care and Use Committees at The University of Kansas Medical Center (Kansas City, KS).

Mice were received single subcutaneous flank injection of 1 × 10^6^ Huh7.5 cells suspended in 100 μL DMEM/Matrigel (1,1 mixture). Tumor growth was monitored by bidimensional measurements using a caliper. Tumor-bearing mice were randomly divided into four groups when tumor size reached 0.5 cm^3^ and immediately receiving pharmacological treatment twice a week via intratumoral injections. RGFP966 (10 mg/kg) was dissolved in DMSO, and diluted in a vehicle of 100 mM sodium acetate (PH 5.4) and 30% (wt/vol) hydroxypropyl-beta-cyclodextrin, with the final DMSO less than 10% (vol/vol). Doxorubicin (2 mg/kg) was dissolved in DMSO and diluted in saline. Vehicles were obtained by identical procedures without drugs. Tumor growth was monitored every 2 days. All mice were sacrificed 2 weeks after the treatment and tumors were removed for further study.

### Human specimens and immunohistochemistry

De-identified human liver specimens from liver explants were obtained from the Liver Center Tissue Bank at the University of Kansas Medical Center. All studies using human tissue samples were approved by the Human Subjects Committee of the University of Kansas Medical Center.

Immunohistochemistry were performed as previously described [18]. After deparaffinization and rehydration, antigen retrieval was achieved by heating in a pressure cooker for 5 min in 10 mM of sodium citrate (pH 6). Peroxidase activity was blocked by incubation in 3% H_2_O_2_ for 10 min. Sections were rinsed three time in PBS/PBS-T (0.1% Tween-20) and incubated in Dako Protein Block (Dako, Agilent Technologies, Santa Clara, CA) for 10 min. After removal of blocking solution, slides were placed into a humidified chamber and incubated overnight with primary antibodies in blocking buffer (3% normal goat serum in PBS) and incubated over night at 4 °C. After washing, slides were covered with SignalStain Boost IHC Detection Reagent (Cell Signaling Technologies, Boston, MA) for 30 min at room temperature. After washing two times with PBS-T, the Substrate-Chromgen Solution (VECTOR NovaRED, Substrate Kit, Vector Laboratories, Burlingame, CA) was applied, slides were incubated 5–10 min and counterstained with Hemtoxylin. Images were acquired using a Nikon Eclipse 80i microscope (Nikon Americas Inc., Melville, NY).

### Isolation and culture of primary human hepatocytes

Primary human hepatocytes [17] were freshly isolated from liver resection specimens by the Cell Isolation Core of the Department of Pharmacology at the University of Kansas Medical Center. All human tissues were obtained with informed consent from each patient, according to ethical and institutional guidelines. The study was approved by the Institutional Review Board at the University of Kansas Medical Center. Cells were isolated using a multi-step collagenase procedure as described in detail [19]. Media consisted of Williams’ Medium E (Invitrogen) supplemented with l-glutamine (2 mM), HEPES (10 mM), insulin (10^− 7^ M), dexamethasone (10^− 7^ M), penicillin (100 U/mL), streptomycin (100 μg/mL) and amphotericin B (0.25 μg/mL). The hepatocytes were brought to a concentration of 0.5 × 10^6^ cells/ml in Williams’ Medium E, as described above, plus 5% bovine calf serum. The hepatocytes were then seeded on collagen coated plates and allowed to attach in a humidified 37 °C, 5% CO_2_ incubator for 12 h and then whole-cell lysates were prepared for western blot.

### Immunofluorescence

For indirect immunofluorescence, cells grown on coverslips were fixed with 4% paraformaldehyde and permeabilized in 0.2% Triton X-100. The coverslips were inverted and touched to 40 μl droplets of blocking buffer (4% goat serum in PBS) on a clean parafilm sheet and incubated for 45 min at room temperature. Cells were then incubated in PBS with mouse anti-p53 (1:100) for 1 h at room temperature. After washing with PBS, coverslips were incubated with Alexa Fluor 488-conjugated goat anti-mouse IgG (1, 5000; Molecular Probes, Waltham, MA, USA) for 1 h in the dark at room temperature. Coverslips were additionally incubated with DAPI for 20 min at room temperature to stain nuclear DNA. Images were acquired using a Nikon Eclipse Ti microscope (Nikon Americas Inc.).

### Real-time PCR

RNA was extracted by using the TRI reagent (Thermo Fisher Scientific, Waltham, MA). cDNA was generated by using the RNA reverse transcription kit (Applied Biosystems, Warrington, UK). Quantitative RT-PCR was performed in a CFX96 real-time system (Bio-Rad, Hercules, CA) using specific sense and antisense primers in 25 μl reaction volumes containing 12.5 μl SYBR Green PCR master mix (Applied Biosystems), 10.5 μl of 1 μmol/l primer stock and 2 μl of cDNA (1:  10 diluted). Primer sequences are presented in Additional file 1: Table S1.

### Western blots

Whole-cell lysates were prepared from cells that had been washed and harvested by centrifugation in PBS. Cell pellets were resuspended in RIPA buffer that contained 50 mM Tris, pH 7.5, 150 mM sodium chloride, 1% NP-40, 0.2% SDS, 0.5% sodium deoxycholate, 0.1 mM EDTA and 1% protease and phosphatase inhibitors (Sigma-Aldrich). Lysates were centrifuged and supernatants were collected. Cell lysates (25 μg) were separated by 10% SDS-PAGE and transferred to polyvinylidene difluoride membranes (Immobilon-P membranes; Millipore, Billerica, MA, USA). Membranes were blocked with blocking buffer (5% skim milk, 0.1% Tween-20 in PBS) for 1 h at room temperature. After incubation with primary antibodies overnight at 4 °C, membranes were then incubated with horseradish peroxidase-conjugated secondary antibodies, detected using the ECL Plus Western Blotting Detection System (Amersham Biosciences, Piscataway, NJ, USA) with the ODYSSEY Fc, Dual-Mode Imaging system (Li-COR, Lincoln, NE).

### Immunoprecipitation

HeLa cells were seeded at 4 × 10^6^ cells/ 10 cm plate and were transiently transfected with 8 μg of HA-SIRT7 and co-transfected with wild type Flag-p53 or various p53 mutant constructs. One day after transfection, cells were incubated in the presence of Trichostatin A (TSA, 10 μM) for 2 h before harvest and were then lysed in the lysis buffer described above. For each immunoprecipitation experiment, 400 μg cell extracts were subjected to immunoprecipitation with 50 μL anti-Flag M2 magnetic beads (Sigma-Aldrich). The immune complexes were analyzed by western-blot.

### In vitro deacetylation assay

HeLa cells were seeded at 4 × 10^6^ cells/10 cm and cultured overnight. Thereafter cells were incubated in the absence or in the presence of H_2_O_2_ (400 μM, 1 h). Extracts were obtained by lysing the cells in RIPA buffer as above. Cell extracts were subjected to immunoprecipitation using the p53 antibody (1C12). Purified p53 was incubated in HDAC buffer (10 mM Tris HCl pH 8.0, 150 mM NaCl, 10% glycerol) with purified recombinant human SIRT7 (0.4 μg), in the presence or absence of NAD^+^ (60 μM) for 1 h at 30 °C. The reactions were resolved on SDS PAGE and analyzed by western-blot.

### Acetylation of endogenous p53

Huh7.5 cells were seeded at 7.5 × 10^5^ cells and transfected with siSIRT7 described above. Three days later, cells were incubated in the presence of TSA for 2 h. Cells were lysed in the RIPA lysis buffer described previously [17]. For doxorubicin treatment, cells were treated with doxorubicin for various times as indicated, or in the presence of sirtinol (Sigma-Aldrich, 20 μM), RSV (100 μM) or C646 (Sigma-Aldrich, 10 μM) for 4 h. Total cell extracts (400 μg protein) were subjected to immunoprecipitation with 4 μg antibody to p53 (1C12). The immune complexes were analyzed by western-blotting with anti-acetyl-K antibody.

### TUNEL assay

For TUNEL assays, cells were fixed with 4% paraformaldehyde at room temperature for 10 min. After a PBS rinse, cells were stained using the DeadEND Fluorometric TUNEL System (Promega, Madison, WI, USA) according to the manufacturer’s instructions. For mouse paraffin-embedded sections, cell death was detected by TUNEL assay kit (In Situ Cell Death Detection Kit, Roche, Indianapolis, IN) according to the manufacturer’s instructions, followed by counterstaining with Fast Green FCF (Acros Organics, NJ). Quantification of TUNEL staining was performed by examining at least five randomly selected fields.

### Antibodies and chemicals

Anti-SIRT7 (D3K5A), anti-SIRT1 (D1D7), anti-SIRT6 (D8D12), anti-FOXO3 (75D8), anti-FOXO1 (C29H4), anti-Acetylated-Lysine (9441), anti-p21(12D1), anti-cleaved caspse3 (5A1E), anti-GADD45 (D17E8), anti-p53 (1C12), anti-p53 (acetyl-K382) (2525) and anti-PARP (9542) were purchased from Cell Signaling Technology (Boston, MA). Anti-GAPDH (FL-335) anti-p53 (DO-1) and anti-NOXA (114C307) were purchased from Santa Cruz Biotechnology (Dallas, TX). Anti-beta actin (AC-15), and anti-Flag (M2) were purchased from Sigma-Aldrich. Anti-p53 (acetyl K320) (06-1283) and anti-p53 (acetyl K373) (06-916) were purchased from Millipore (Burlington, MA). Anti-p53 (acetyl K373) (ab62376), Anti-p53 (acetyl K382) (ab75754), anti-NOXA (ab13654) were purchased from Abcam (Cambridge, MA). Resveratrol, Trichostatin A (TSA), C646, MG132, sirtinol and doxorubicin were purchased from Sigma-Aldrich. NAD^+^ was purchased from Cayman Chemical (Ann Arbor, MI). rSIRT7 was purchased from SignalChem (Richmond, BC, Canada). SP600125, LY294002 were purchased from Cell Signaling Technology. SB203580 was purchased from AdipoGen (San Diego, CA, USA). FR180204 was purchased from Calbiochem (Billerica, MA, USA). RGFP966 was purchased from SelleckChem (Houston, TX).

### Statistical analysis

Data are presented as mean ± sem. Statistical significance between groups was calculated by using one-way ANOVA followed by Turkey’s test. Statistical significance between two groups was calculated by 2-tailed unpaired Student’s t-test. Variance between groups met the assumptions or the appropriate test. Unless otherwise stated, a *P*-value of < 0.05 was considered statistically significant. The Kaplan–Meier method was used to estimate the survival rates for SIRT7 expression. Equivalences of the survival curves were tested by log-rank statistics.

## Results

### Elevated SIRT7 expression in human HCC positively correlates with TACE-resistance

**Fig. 1:**
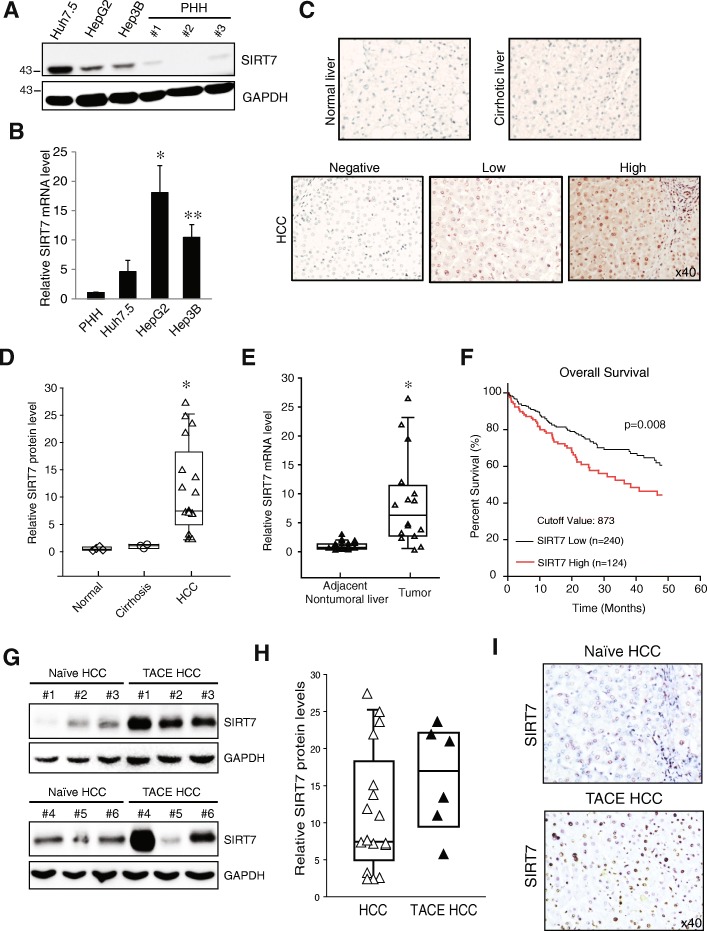
SIRT7 expression in human HCC. Protein (**a**) and mRNA (**b**) levels of SIRT7 from isolated primary human hepatocyte (PHH) and HCC cell lines. **c** Representative IHC staining of SIRT7 in normal, cirrhotic and HCC liver sections. **d** Quantitative analysis of SIRT7 protein levels in normal (*n* = 4), cirrhotic (*n* = 3) and HCC (*n* = 17). The data is presented as fold increase compared with normal liver and was normalized to GAPDH. *P < 0.05 vs normal, Student’s t-test. **e** RT-PCR analysis of SIRT7 mRNA levels in 20 paired nontumoral and HCC tissues. *P < 0.05, Student’s t-test. **f** Kaplan–Meier analysis of overall survival in 364 liver cancer patients based on SIRT7 expression. **g** and **h** Western blot analysis of SIRT7 protein levels (**g**) and quantitative analysis of SIRT7 protein levels in HCC as in D (*n* = 17) and TACE treated HCC (*n* = 6) (**h**). The data is presented as fold increase compared with normal liver and was normalized to GAPDH. **i** IHC staining of SIRT7 in Naïve and TACE treated HCC tissues

We first analyzed SIRT7 expression patterns in three HCC cell lines (Huh7.5, Hep3B and HepG2) and primary human hepatocytes (PHH). SIRT7 was almost undetectable in PHH, whereas high SIRT7 expression was observed in all three HCC cell lines at both the protein and mRNA levels (Fig. 1a and b). We further examined SIRT7 expression in normal, cirrhotic and HCC tissue by using immunohistochemistry (IHC). SIRT7 immunoreactivity was graded as negative (score 0), low (low to medium staining, scores 1-2), and high (strong staining, score 3). We found that in both normal and cirrhotic liver sections, SIRT7 staining was undetectable. However, 12 out of 17 (70.6%) HCC samples were SIRT7 positive. Among the 12 positive HCC samples, 6 showed low SIRT7 and 6 showed strong SIRT7 staining (Fig. 1c). SIRT7 expression level was further analyzed in these samples by western blot. We found significantly higher SIRT7 levels in HCC compared with normal and cirrhotic liver (Fig. 1d, Additional file 2: Figure S1). We also compared mRNA expression in 20 pairs of frozen HCC and adjacent nontumoral liver tissues by qPCR. SIRT7 mRNA levels were significantly upregulated in HCC compared with adjacent nontumoral liver tissues (Fig. 1e), suggesting that SIRT7 overexpression in HCC is regulated in a transcription-dependent manner. Correlative analysis of SIRT7 mRNA levels with clinicopathologic features also suggested significant association between increased SIRT7 expression and vascular invasion (*p* < 0.05) (Table 1). We analyzed previously published HCC data sets [20] that contain SIRT7 expression and detailed survival information; the Kaplan–Meier analysis revealed that patients with high SIRT7 expression levels in liver cancer tissues had significantly shorter overall survival rates than those with low SIRT7 expression (*p* = 0.008, Fig. 1f).

**Table 1:** Correlative analysis of SIRT7 mRNA levels with clinicopathological features. High tumoral SIRT7 expression was considered > 2-fold up-regulation relative to the adjacent non-tumoral liver. Correlations between SIRT7 and individual clinicopathologic parameters were evaluated using a nonparametric Fisher’s Exact Test

Clinicopatholigic Parameters	Number of Specimens	SIRT7 expression (Tumor/Nontumoral)		*P* value
		low	high	
Sex				1.000
Female	6	2	4	
Male	11	4	7	
Age(mean ± SD)		62.2 ± 4.7	62.7 ± 8.1	0.9598
Tumor size				0.6000
> 3 cm	11	3	8	
< 3 cm	6	3	3	
Multiple Tumor				0.2801
Yes	12	3	9	
No	5	3	2	
Vascular Invasion				0.0498
Yes	9	1	8	
No	8	5	3	
TACE Treatment				0.2801
Yes	12	3	9	
No	5	3	2	
Recurrence				0.5147
Yes	2	0	2	
No	15	6	9	

We next examined the potential role of SIRT7 in TACE-resistance. We compared SIRT7 expression levels in treatment naïve HCC that never received TACE treatment (Naïve HCC) and HCCs that were treated with TACE but recurred after therapy (TACE resistant). We found 5 out of 6 (83.3%) TACE-resistant HCCs showed elevated SIRT7 protein expression levels (Fig. 1g). TACE-resistant HCC showed more than 2-fold elevation of SIRT7 protein level when compared with overall HCC (Fig. 1h). IHC staining indicated strong nuclear staining of SIRT7 compared with naïve HCC (Fig. 1h). These data suggest that SIRT7 may play a role in regulating HCC proliferation and chemosensitivity.

### SIRT7 regulates doxorubicin induced cell death in HCC cell lines

**Fig. 2:**
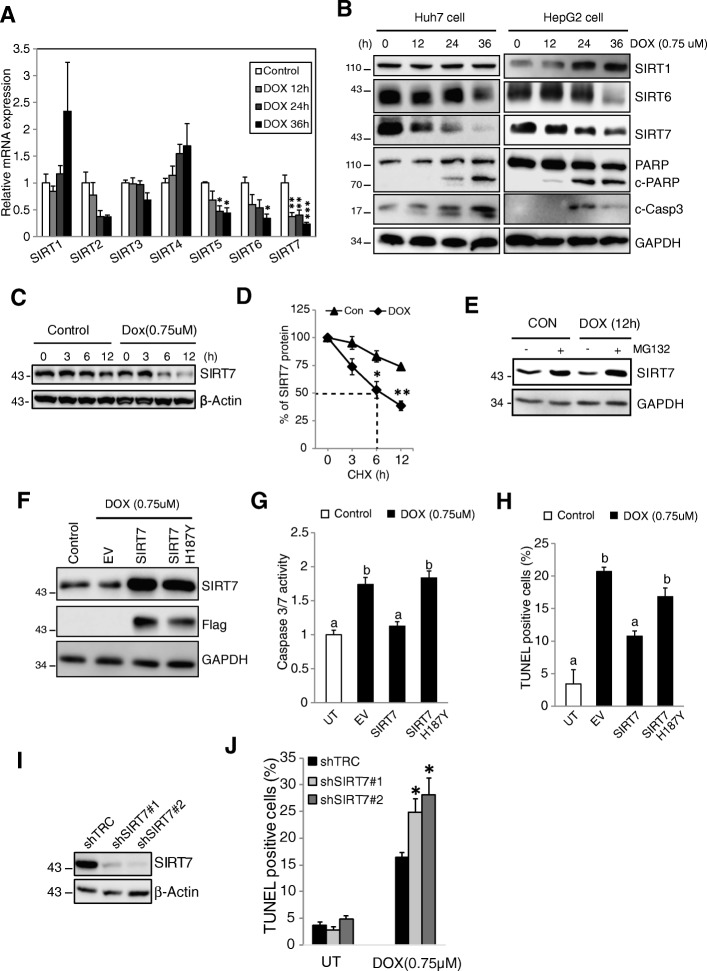
SIRT7 is critical in determining doxorubicin induced cell death. **a** Huh7.5 cells were untreated (Control) or treated with doxorubicin (DOX, 0.75 μM) for 36 h. Cells were harvested at various time points as indicated. mRNA levels of SIRT1-7 were evaluated by RT-PCR. *P < 0.05, **P < 0.01, ***P < 0.001 vs Control, one way ANOVA. **b** HepG2 and Huh7.5 cells were treated with doxorubicin for various time and protein levels were evaluated by western blot. **c** and **d** SIRT7 protein half-life in Huh7.5 cells either untreated (Con) or treated with doxorubicin in the presence of cycloheximide (CHX, 100 μM). *P < 0.05, *P < 0.01 vs Con, Student’s t-test. **e** SIRT7 protein level in Huh7.5 cells either untreated (CON) or treated with doxorubicin for 12 h in the absence or presence of the proteasome inhibitor MG132 (50 μM). **f**-**h** Huh7.5 cells were untransfected (Control) or transfected with empty vector (EV), SIRT7 or SIRT7 187HY for 24 h, followed by doxorubicin treatment for another 36 h. Protein expression levels were evaluated by western blot (**f**) and cell death were evaluated by caspase 3/7 activity (**g**) and TUNEL assay (**h**). Values with different superscripts are significantly different from each other (*p* < 0.05, one way ANOVA). **i** and **j** Huh7.5 cells were treated with scramble shRNA (shTRC) or shRNA targeting SIRT7 (shSIRT7#1 and shSIRT7#2) for 72 h, SIRT7 levels were evaluated by western blot (**i**). Cells were then treated with doxorubicin for 36 h and cell death were evaluated by TUNEL assay (**j**). *P < 0.05 vs shTRC/DOX, one way ANOVA. Data are representative of three independent experiments. Graphs show mean ± SEM of at least three independent experiments

To further explore the role of SIRT7 in therapy sensitivity of HCC, we treated Huh7.5 and HepG2 cells with doxorubicin (0.75 μM) and examined changes of SIRT7 expression. Doxorubicin treatment resulted in significant downregulation of SIRT7 mRNA and protein levels as early as 12 h (Fig. 2a, b). Immunofluorescence indicated doxorubicin decreased global SIRT7 intensity from 24 h post-treatment (Additional file 2: Figure S2A). Downregulation of SIRT7 was associated with doxorubicin induced cell death as evidenced by PARP cleavage and caspase 3 activation (Fig. 2b). We next measured SIRT7 protein stability in the presence of cycloheximide (CHX). As shown in Fig. 2c and d, doxorubicin decreased the half-life of SIRT7 and the proteasome inhibitor MG-132 increased the amount of SIRT7 after doxorubicin (Fig. 2e). This suggests that an active process of SIRT7 proteolysis is induced by doxorubicin and the decrease in protein level results both from changes in mRNA expression and protein stability. We also observed that doxorubicin induced a decrease of SIRT6 mRNA and protein levels, however, in contrast to SIRT7 this decrease was only observed 36 h after treatment (Fig. 2a, b).

The mitogen-activated protein kinase (MAPK) cascade is a key downstream pathway for doxorubicin-induced signaling events [21] and has also been implicated as a regulator of SIRT7 stability [10, 22]. We found that both JNK (SP600125) and p38 (SB203580) inhibitors diminished doxorubicin induced decreases of SIRT7 mRNA and protein (Additional file 2: Figure S2B, S2C). To further confirm whether JNK and p38 are responsible for SIRT7 degradation, we overexpressed variants of MAPKs and monitored the SIRT7 signal by immunofluorescence. Overexpression of JNK1 or p38 decreased SIRT7 in Huh7.5 cells (Additional file 2: Figure S2D). Immunoprecipitation experiments also demonstrated direct binding of SIRT7 to JNK1 and p38 (Additional file 2: Figure S2E). These data suggested that that JNK1 and p38 paly vital roles in the SIRT7 decrease during doxorubicin treatment.

To determine whether downregulation of SIRT7 is required for doxorubicin induced apoptosis, Flag-tagged SIRT7 and an inactive SIRT7 H187Y mutant were overexpressed in Huh7.5 cells followed by treatment with doxorubicin (Fig. 2f). SIRT7 overexpression almost completely abolished doxorubicin-induced caspase 3/7 activation and cell death (Fig. 2g, h). Conversely, we found that knockdown of SIRT7 significantly increased doxorubicin induced cell death (Fig. 2i-j). These data indicate that SIRT7 plays a critical role in determining cell fate in response to doxorubicin and this effect requires SIRT7 deacetylase enzyme activity.

### SIRT7 regulates chemosensitivity primarily through p53

**Fig. 3:**
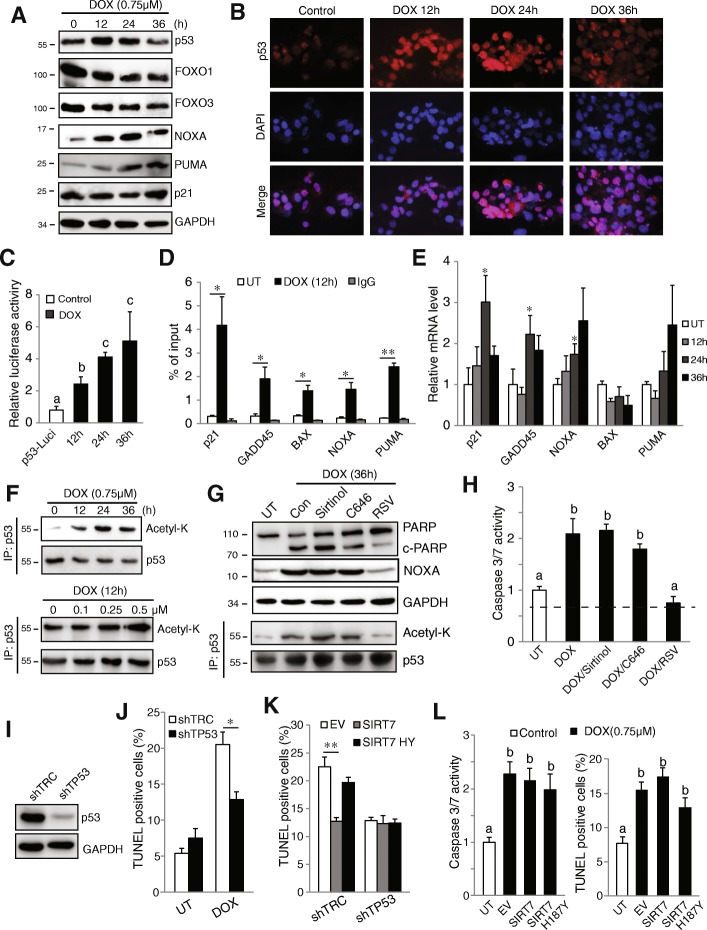
SIRT7 regulates doxorubicin induced cell death via p53. **a** Huh7.5 cells were treated with doxorubicin (DOX) for various time. Protein levels were evaluated by western blot by using antibodies as indicated. **b** Immunofluorescence for p53 (red) in cells treated with doxorubicin as in A. **c** Huh7.5 cells were transfected with luciferase reporter plasmid (p53-Luci) containing p53-binding consensus sequence, luciferase activity was measured in the absence or presence of doxorubicin treatment at various of time points. Values with different superscripts are significantly different from each other (*p* < 0.05, one way ANOVA). **d** Huh7.5 cells were untreated (UT) or treated with doxorubicin for 12 h. ChIP assay was performed with anti-p53 antibody. *P < 0.05, **P < 0.01 vs UT, Student’s t-test. **e** RT-PCR analysis of p53 target gene mRNA levels in cells untreated (UT) or treated with doxorubicin. *P < 0.05, vs UT, one way ANOVA. **f** Huh7.5 cells were treated with doxorubicin for various of time or dose as indicated, p53 were immunoprecipitated from cell lysates and acetylation levels of p53 were evaluated by western blot. **g** and **h** Huh7.5 cells were treated with doxorubicin in the absence (UT) or presence DMSO (Con), Sirtinol (20 μM), C646 (25 μM) or RSV (100 μM) for 36 h, protein levels were evaluated by western blot (**g**) and cell death were measured by caspase3/7 activity (**h**). Values with different superscripts are significantly different from each other (*p* < 0.05, one way ANOVA). **i** Huh7.5 cells were treated with scramble (shTRC) or shRNA targeting p53 (shTP53) for 72 h, p53 levels were evaluated by western blot. **j** Cells in I were untreated (UT) or treated with doxorubicin for 36 h and cell death were evaluated by TUNEL assay. *P < 0.05 vs shTRC/DOX, Student’s t-test. **k** Cells in I were transfected with empty vector (EV), SIRT7 or inactive SIRT7 H187Y for 24 h followed by doxorubicin treatment for 36 h, Cell death were evaluated by TUNEL assay. **P < 0.01 vs EV, Student’s t-test. **l** Hep3B cells were transfected with empty vector (EV), SIRT7 or inactive SIRT7 H187Y for 24 h, cell were left untreated (Control) or treated with doxorubicin for 36 h. Cell death were evaluated by caspase3/7 activity and TUNEL assay. Values with different superscripts are significantly different from each other (*p* < 0.05, one way ANOVA). Data are representative of three independent experiments. Graphs show mean ± SEM of at least three independent experiments

To determine the potential SIRT7 target that is responsible for regulating doxorubicin-induced apoptosis, we evaluated multiple proteins that are known to induce apoptosis, including p53 [23], FOXO3 [24] and FOXO1 [25]. In Huh7.5 cells, doxorubicin treatment resulted in a slight decrease of FOXO1 and FOXO3 protein expression, whereas p53 protein was significantly elevated. There was a strong correlation between expression levels of p53 and its targets NOXA and PUMA (Fig. 3a). Immunofluorescence staining also indicated that doxorubicin treatment significantly increased p53 intensity 12 h post-treatment and this p53 was primarily localized in the nucleus (Fig. 3b). Nuclear localization of p53 was associated with p53 transcriptional activity as shown by a luciferase reporter plasmid containing the p53-binding consensus sequence [26] (Fig. 3c). To investigate whether nuclear p53 was bound to specific target gene promoters, a ChIP assay was performed. As indicated in Fig. 3d, 12 h of doxorubicin treatment significantly increased p53 binding to its target genes. Consistent with this, RT-qPCR results indicated that mRNA levels of p21, GADD45 and NOXA were significantly increased following treatment (Fig. 3e).

Since acetylation is an established mechanism of regulation of p53-dependent apoptosis and senescence in many cell types [27, 28], we measured p53 acetylation after doxorubicin treatment (Fig. 3f). We found that doxorubicin treatment induces p53 acetylation in a time- and dose-dependent manner. To examine whether acetylation of p53 is required for doxorubicin induced apoptosis, we treated cells with doxorubicin in the presence of a multispecific SIRT inhibitor (Sirtinol), a SIRT activator (resveratrol), or a p300 acetyltransferase inhibitor (C646). Figure 3g demonstrates that sirtinol increases p53 acetylation and doxorubicin induced PARP cleavage. Inhibition of the acetyl-transferase p300 had only minor effects on p53 acetylation and cell death. In contrast, resveratrol (RSV), a well-known SIRT activator, almost completely abolished p53 acetylation and doxorubicin induced NOXA expression, PARP cleavage and caspase 3/7 activity (Fig. 3g, h).

We next assessed whether SIRT7 regulates doxorubicin induced cell death through p53. We knocked down p53 in Huh7.5 cell (Fig. 3i) and treated cells with doxorubicin. Knockdown of p53 largely decreased doxorubicin induced apoptosis (Fig. 3j). We further overexpressed SIRT7 or inactive SIRT7 HY in either control or p53 knockdown cells and treated them with doxorubicin. Both overexpression of WT SIRT7 in control (shTRC) cells or knockdown of p53 by itself prevented doxorubicin induced cell death, however, no additive effects were observed when we overexpressed SIRT7 in p53 knockdown cells. After p53 knockdown, there was no difference in apoptosis rate between WT and inactive SIRT7 overexpression (Fig. 3k). To further confirm these findings, we used Hep3B cell lines which do not express p53 [29]. In these cells, SIRT7 overexpression failed to protect against doxorubicin induced caspase3/7 activation or cell death (Fig. 3l). These data indicate that SIRT7 regulates doxorubicin induced apoptosis primarily through a p53-dependent mechanism.

### SIRT7 interacts with and deacetylase p53 both in vitro and in vivo

**Fig. 4:**
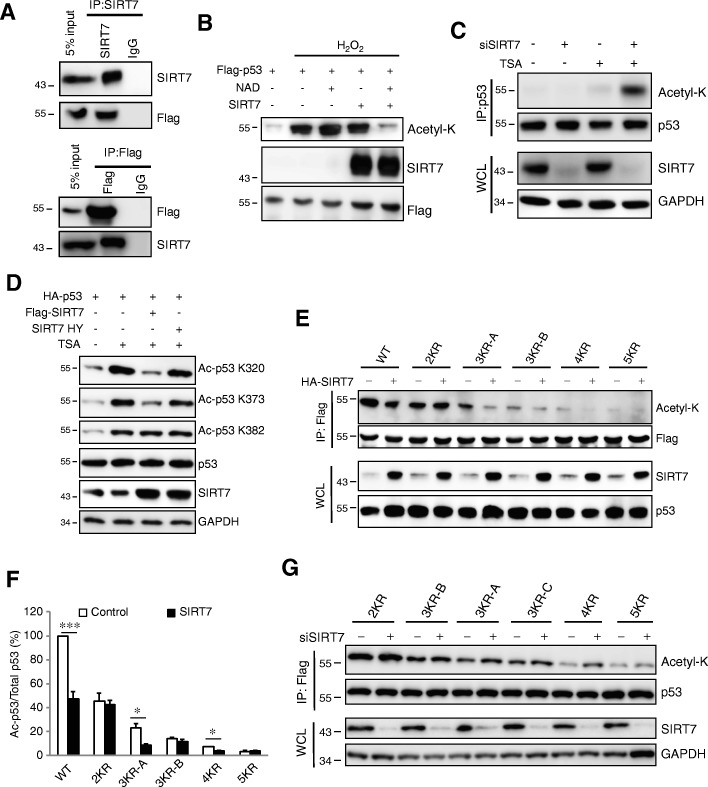
SIRT7 deacetylates p53 at K320 and K373. **a** Flag-p53 were overexpressed in Huh7.5 cells, Endogenous SIRT7 or Flag-p53 were immunoprecipitated from cell lysates and the immune complexes were assessed for the presence of p53 or SIRT7 by Western blot. **b** Deacetylation of p53 by SIRT7 in vitro. The Flag-p53 was purified from HeLa cells treated with or without H_2_O_2_ (400 μM, 1 h). Purified p53 was incubated in the presence or absence of rSIRT7 with or without NAD^+^. Acetylated and total amount of p53 were assessed by western blot with anti-acetyl-K and p53. **c** Deacetylation of p53 by SIRT7 in cells. Huh7.5 cells were transfected with siSIRT7 for 72 h and then incubated in the presence of TSA (5 μM) for 2 h. Cells were lysed and cell extracts were subjected to immunoprecipitation with antibody to p53. Acetylated p53 was analyzed by western blot with anti-acetyl-K and total protein was evaluated in whole cell lysates (WCL). **d** Huh7.5 cells were co-transfected with HA-p53 with Flag-SIRT7 or Flag-SIRT7 HY for 24 h, cells were then incubated with TSA for 2 h. Cells were lysed and cell extracts were subjected to western blot. **e** Huh7.5 cells were co-transfected wild type Flag-p53 (WT), Flag-p53 K320,373R (2KR), Flag-p53 K320,381,382R (3KR-A), Flag-p53 K120,320,373R (3KR-B), Flag-p53 K372,373,381,382R (4KR), Flag-p53 K120,372,373,381,382R (5KR) with or without HA-SIRT7 for 24 h and then incubated in the presence of TSA for 2 h. Cells were lysed and cell extracts were subjected to immunoprecipitation with magnetic beads to Flag. Acetylated p53 was analyzed by western blot. **f** Quantification of acetylation levels of p53 mutants in the absence (Control) or presence of SIRT7. Graphs show mean ± SEM of three independent experiments. *P < 0.05, ***P < 0.001 vs Control, Student’s t-test. **g** Huh7.5 cells were transfected with siSIRT7 for 48 h and overexpressed 2KR p53, 3KR-A, 3KR-B, Flag-p53 K373,381,382R (3KR-C), 4KR, 5KR for 24 h, acetylated p53 was analyzed as in (**d**). Data are representative of three independent experiments

Multiple SIRT family members have been reported to deacetylate p53 [8, 23, 30–32]. We thus investigated whether SIRT7 deacetylates p53 as well. We overexpressed Flag-p53 in Huh7.5 cells and immunoprecipitated with either Flag or SIRT7 antibodies. This indicated interaction between these two proteins (Fig. 4a). Immunofluorescence also indicated co-localization of SIRT7 and p53 in the nucleus (Additional file 2: Figure S3A). We next examined whether SIRT7 deacetylates p53. Flag-p53 was expressed in HeLa cells and cells were treated with hydrogen peroxidase (H_2_O_2_) to induce acetylation of p53 [33]. We then purified this acetylated p53 and incubated it in vitro with active human recombinant SIRT7 (rSIRT7). As reported, H_2_O_2_ induced acetylation of p53 which became de-acetylated only after the simultaneous addition of rSIRT7 and NAD^+^ (Fig. 4b). To examine the role of SIRT7 in p53 deacetylation in cells, we knocked down SIRT7 in Huh7.5 cells that had been incubated with the Class I and II HDAC inhibitor TSA and measured p53 acetylation. SIRT7 knock down alone had no effect on p53 acetylation. Treatment with TSA alone slightly increased p53 acetylation but incubation of siSIRT7 treated cells with TSA led to a dramatic increase in p53 acetylation (Fig. 4c). These results suggest that both SIRT7 and Class I and II HDACs contribute to p53 deacetylation within cells.

To further investigate SIRT7-dependent deacetylation, Flag-p53 were co-transfected with HA-SIRT7 or inactive form and evaluated p53 acetylation by using antibodies that recognize acetylation sites of p53 [28]. As shown in Fig. 4d, in the presence of TSA, SIRT7 but not its inactive form decreased p53 acetylation at K320 and K373, but not at K382. To investigate if other sites also deacetylated by SIRT7, we generate p53 KR mutants in which the six known acetylated lysines [28] including K320, K373 were replaced with arginine. We transfected p53 WT and mutants with SIRT7 and compared acetylation levels (Fig. 4e and Additional file 2: Figure S3B). SIRT7 overexpression significantly decreased acetylation of WT p53, but failed to decrease acetylation of the p53-double KR mutants (K320, 373R), triple-KR mutants (K120, 320, 373R, 3KR-B) and 5KR mutants (K120, 320, 373, 381 and 382R) in which K320 and K373 were replaced with arginine (Fig. 4e, f). Mutation of either K320 nor K373 alone (Additional file 2: Figure S3B), or triple-KR mutants (K320, 381, 382R, 3KR-A) and quadruple-KR mutant (K372, 373, 381, 382R, 4KR) mutants that only including single arginine of K320 or K373 was not sufficient block SIRT7 dependent deacetylation of p53 (Fig. 4e, f). These data suggested that SIRT7 deacetylates p53 at both K320 and K373. To further confirm these finding, we transfected p53 WT and mutants in control or SIRT7 knockdown cells and compared acetylation levels. As shown in Fig. 4g, KR mutants that include K320 and K373 (2KR, 3KR-B, 5KR) did not show any increase of acetylation after SIRT7 knockdown, whereas mutants including either single arginine of K320 or K373 had an increase of acetylation after SIRT7 knockdown (Fig. 4g). We finally evaluated the cellular localization of the p53 KR mutants. We found that the 5KR mutant shows a partially cytosolic distribution, however, the 2KR mutant had the same distribution pattern as wild-type p53 (Additional file 2: Figure S3C). Taken together, these data indicated that in HCC, SIRT7 interacts with p53 and induces p53 deacetylation at K320 and K373.

### SIRT7 suppresses p53-dependent NOXA expression

**Fig. 5:**
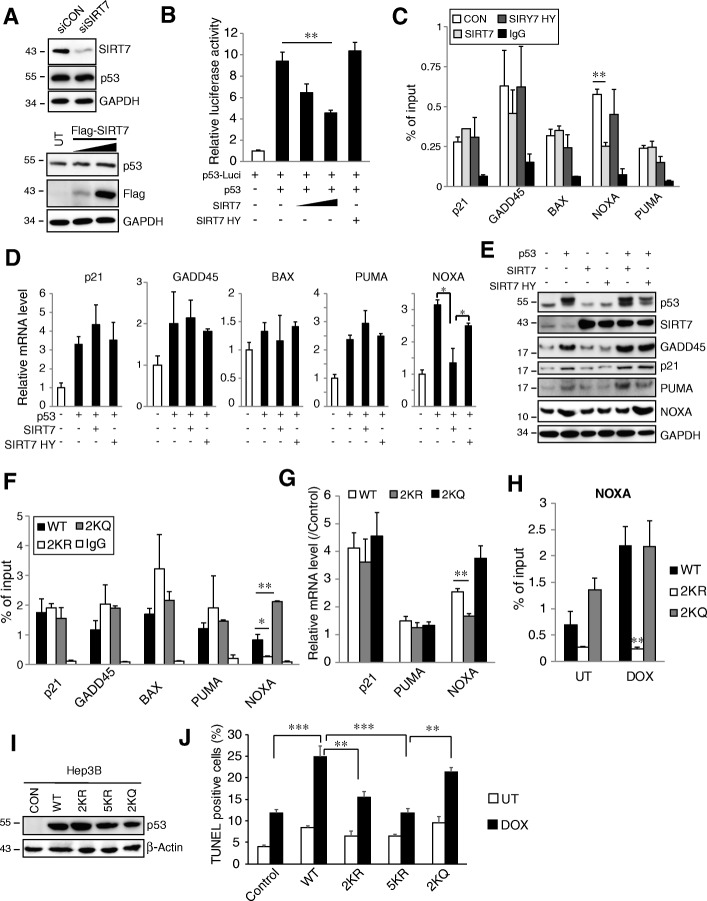
SIRT7 regulates p53 mediated NOXA expression. **a** Western blot analysis of p53 protein levels Huh7.5 cell after SIRT7 knockdown (upper) or overexpression (lower). **b** Luciferase activity from Huh7.5 were transfected with luciferase reporter plasmid containing p53-binding consensus (p53-Luci) with or without p53 in the absence or presence of SIRT7 or SIRT7 H187Y (SIRT7 HY). **P < 0.01, one way ANOVA. **c** Huh7.5 cells were transfected with Flag-p53 in the absence (CON) or presence of SIRT7 or SIRT7 HY. ChIP assay was performed with anti-Flag antibody. **P < 0.01, Student’s t-test. **d** and **e** RT-PCR analysis of mRNA levels (**d**) and western blot analysis of protein levels (**e**) in Huh7.5 cells were untransfected or transfected with p53 in the absence or presence of SIRT7 or SIRT7 HY. *P < 0.05, Student’s t-test. **f** Huh7.5 cells were transfected with Flag-p53 (WT), Flag-p53 K320,373R (2KR), or Flag-p53 K320,373Q (2KQ). ChIP assay was performed with anti-Flag antibody. *P < 0.05, **P < 0.01, one way ANOVA. **g** RT-PCR analysis of mRNA levels in cells as in F. **P < 0.01, one way ANOVA. **h** Huh7.5 cells were transfected with WT, 2KR or 2KQ p53 for 24 h, cell were left untreated (UT) or treated with doxorubicin for 24 h, ChIP assay was performed with anti-Flag antibody. **P < 0.01 vs WT/DOX, one way ANOVA. **i** and **j** Hep3B cells were transfected with WT, 2KR, 2KQ or 5KR (K120,372,373,381,382R) for 24 h and treated with doxorubicin for 36 h, p53 expression were evaluated by western blot (**i**) and cell death were evaluated by TUNEL assay (**j**). **P < 0.01, ***P < 0.001, one way ANOVA. Data are representative of three independent experiments. Graphs show mean ± SEM of at least three independent experiments

We further examined SIRT7-dependent regulation of p53 transcriptional activity. Expression or knockdown of SIRT7 in Huh7.5 cells did not change protein abundance of p53 (Fig. 5a). However, WT SIRT7, but not the inactive form, significantly decreased p53 transcriptional activity (Fig. 5b). To assess the effects of SIRT7 on the binding of p53 to its target promoters we performed ChIP assays. As shown in Fig. 5c SIRT7, but not inactive SIRT7, significantly reduced p53 binding to the NOXA promoter but had nearly no effect on binding to promoters for p21, GADD45, BAX and PUMA. Consistent with these ChIP results, we observed that SIRT7 completely abolished the p53 overexpression-enhanced portion of NOXA mRNA and protein expression (Fig. 5d, e). To further examine whether SIRT7 regulates NOXA expression through deacetylation of p53, we evaluated promoter binding of the 2KR mutant missing the SIRT7 deacetylation sites K320 and K373 as well as an acetylation mimetic form of p53 in which K320 and K373 were mutated to glutamine (2KQ). As shown in Fig. 5f, the 2KR mutant showed a defect of binding to the NOXA promoter compared with WT protein. The 2KQ mutant, in contrast, showed significantly higher binding affinity to the NOXA promoter. Compared with WT p53, the 2KR mutant showed nearly no induction of NOXA mRNA transcription but the 2KQ almost doubled NOXA transcription (Fig. 5g). There was no difference of promoter binding and gene transcription between WT, 2KR and 2KQ on p21, GADD45, BAX and PUMA (Fig. 5f, g). In response to doxorubicin treatment, the 2KR mutant showed nearly no induction of NOXA promoter binding whereas WT and 2KQ showed similar binding ability (Fig. 5h).

Finally, we examined whether acetylation at K320 and K373 is required for doxorubicin induced cell death in HCC. We used p53-null Hep3B cells mentioned above and overexpressed p53 WT, 2KR, 5KR and 2KQ mutants and evaluated cell death by TUNEL assay (Fig. 5i-j). As shown in Fig. 5j, restorative overexpression of WT p53 or the mutants increased cell death at baseline. WT p53 significantly increased doxorubicin sensitivity but this effect was not present in both 2KR and 5KR mutants. Similar results were also observed in p53 knockdown Huh7.5 cells. Doxorubicin-induced cell death increased when p53 was restored with the WT or 2KQ protein. However, the 2KR and 5KR mutants did not support an increase in doxorubicin induced cell death (Additional file 2: Figure S3D).

### Inactive SIRT7 enhances doxorubicin toxicity both in vitro and in vivo

**Fig. 6:**
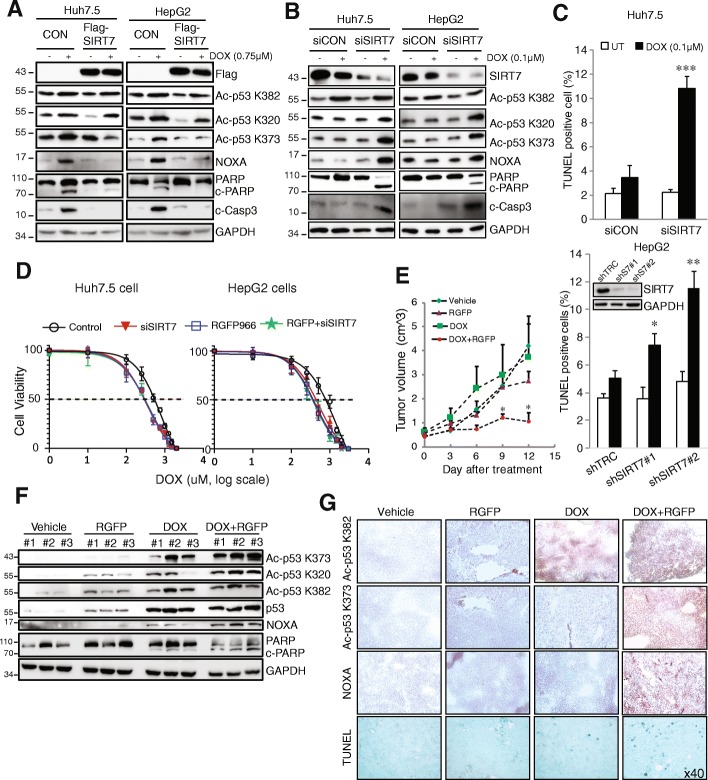
Inhibition SIRT7 enhances doxorubicin toxicity. **a** HepG2 and Huh7.5 cells were transfected with Flag-SIRT7 for 24 h and treated with doxorubicin (0.75 μM) for 36 h. Protein levels were evaluated by western blot. **b** and **c** HepG2 and Huh7.5 cells were transfected with siSIRT7 or shSIRT7 as indicated for 72 h, cells were left untreated (UT) or treated with doxorubicin (0.1 μM) for 36 h. Protein levels were evaluated by western blot (**b**) and cell death were evaluated by TUNEL assay (**c**). *P < 0.05, **P < 0.01, ***P < 0.001 vs UT, Student’s t-test. The inset shows SIRT7 knockdown efficiency in HepG2 cells. **d** Concentration responsiveness curves of doxorubicin in Huh7.5 and HepG2 cells were untreated (Control) or treated with siSIRT7 for 72 h, followed by doxorubicin treatment in the absence or presence of RGFP966 (10 μM) for 36 h. Determination of the inhibitory concentration (IC50) values were obtained by log-linear interpolation of data points. Dot line indicates IC50 values of doxorubicin in various conditions. **e** Tumor growth curves of tumor bearing NSG mice were received Vehicle, RGFP966 (RGFP, 10 mg/kg), doxorubicin (2 mg/kg) or combination (DOX + RGFP) treatment for 2 weeks. **f** Western lot analysis of protein levels in tumor that receiving different treatment. **g** IHC staining analysis in tumors as in (**e**). Data are representative of three independent experiments. Graphs show mean ± SEM

The above studies demonstrate that overexpression SIRT7 greatly decreased doxorubicin induced cell death. We thus further examined whether SIRT7 regulates therapy sensitivity through deacetylation of p53 and its effect on NOXA expression. We overexpressed SIRT7 in Huh7.5 and HepG2 cells and treated cells with doxorubicin. In both cell types, without SIRT7 overexpression, doxorubicin induced dramatic increases of acetylation of p53 at K320, 373 and K382, elevated NOXA protein level, which associated with PARP cleavage and caspase 3 activation (Fig. 6a). Overexpression of SIRT7 decreased the doxorubicin induced K320, K373 acetylation of p53, but showed no effect on K382 acetylation level. More importantly, we observed decreased NOXA expression and less activation of cell death pathways as evidenced by abolished PARP cleavage and caspase 3 activity (Fig. 6a). These data suggest that knockdown SIRT7 might increase doxorubicin toxicity in HCC cells. To test this, we knocked down SIRT7 in Huh7.5 and HepG2 cells and treated the cells with a low dose of doxorubicin (0.1 μM). This dose of doxorubicin only mildly decreases SIRT7 expression and did not induce detectable PARP cleavage, caspase 3 activation and TUNEL positivity in control cells (Fig. 6b, c). However, this knockdown of SIRT7 significantly increased p53 K320, K373 acetylation, NOXA expression, PARP cleavage, caspase 3 activation and TUNEL positivity (Fig. 6b, c).

We further tested whether inhibition of SIRT7 activity could increase doxorubicin toxicity. By using the pharmacological HDAC 3-selective inhibitor RGFP966 [34–36], which inhibits SIRT7 deacetylation activity in vitro (Additional file 2: Figure S4), we observed that RGFP966 significantly increased doxorubicin IC50 in both Huh7.5 and HepG2 cells (2.8- and 3.5-fold, respectively). More interestingly, we found that knockdown of SIRT7 also increased doxorubicin sensitivity in both cell types, however, there was no addictive effects with combination treatment (Fig. 6d). This suggests that SIRT7 could be a primary target of the RGFP966 inhibitor in response to doxorubicin in vitro. We thus tested the effect of RGFP966 on doxorubicin toxicity in vivo by using xenograft mouse model. Doxorubicin (2 mg/kg) and RGFP966 (10 mg/kg) treatment alone had no effects of tumor growth whereas the combination treatment significantly lowered tumor growth rate (Fig. 6e). Western blot examination revealed that tumors receiving doxorubicin and RGFP966 treatment showed a significantly higher level of p53 acetylation at K320 and K373, higher NOXA protein expression, more PARP cleavage and greater caspase 3 cleavage (Fig. 6f). Doxorubicin treatment induced p53 acetylation at K382 but there was no further increase in the combination treatment groups (Fig. 6f). Consistent with these findings, IHC staining also showed that doxorubicin and doxorubicin in combination with the inhibitor equally induced p53 acetylation at K382, however, there was a significantly increased intensity of p53 acetylation at K373, increased NOXA staining, and increased TUNEL positive cells in tumors receiving the combination treatment (Fig. 6g).

## Discussion

SIRT7 is possibly the least understood mammalian sirtuin, but its activity is important for human disease, particularly cancer. Increased SIRT7 expression is observed in many human cancers and growing evidence suggests that it plays important functions in fundamental cellular programs that impact oncogenic transformation and tumor biology [13, 37–39]. SIRT7 associates with chromatin where it selectively deacetylates H3K18, an emerging epigenetic biomarker of aggressive tumors and poor clinical outcome in patients with cancer [40–42]. This deacetylation also regulates tumor suppressive gene expression programs to promote oncogenic transformation [12]. Knockdown of SIRT7 influences the cell cycle and causes a significant increase in the proportion of liver cancer cells that remain in the G1/S phase [12]. In epithelial prostate carcinomas, high SIRT7 levels are associated with aggressive cancer phenotypes, metastatic disease and poor patient prognosis [14]. SIRT7 expression is also upregulated in a large cohort of HCC patents and SIRT7 expression is associated with increased tumor grade [13]. Consistent with these findings, we also observed that both SIRT7 mRNA and protein levels were significantly upregulated in most HCC tissues compared with adjacent nontumoral liver tissues. More importantly, SIRT7 expression significantly correlated with poor overall survival rate in 364 liver cancer cases. We also found that SIRT7 expression was low in normal liver and in primary human hepatocytes. These data suggest that SIRT7 may act as an oncogene in HCC development.

The p53 response pathway is the second most frequently defective pathway in HCC and is altered in at least half of HCC patients [43]. Disruption of the p53 pathway in HCC can either occur by mutations of p53 itself, by alterations in pathways that regulate protein stability such as interaction with its specific inhibitor MDM2 [44], and post-translational modifications such as protein acetylation which lead to enhanced activity. In cardiomyocytes, SIRT7 deacetylates p53 and suppresses p53-dependent cell death in response to oxidative stress [8]. We found that in HCC, SIRT7 interacts with p53. This interaction does not affect p53 protein abundance but it deacetylates p53 and suppresses its transcriptional activity. In addition, we found high SIRT7 expression in the majority of HCC patients. Our findings thus provide evidence that SIRT7 serves as a critical factor that inactivates p53 in those HCCs which express p53 thus contributing to tumor progression. It is unclear whether p53 is the only SIRT7 target gene responsible for HCC proliferation. Notably, Kim et al. [13] observed that knockdown SIRT7 in Hep3B cells (which do not express p53 [45]) also resulted in significantly impaired cell proliferation, suggesting p53 is not the only target.

Defects in the p53 pathway in HCC also contribute to resistance to anticancer therapeutics [46]. In the context of doxorubicin treatment, we showed that SIRT7 controls therapy sensitivity primary through p53. Overexpression of SIRT7 largely abolished doxorubicin induced cell death in both Huh7 and HepG2 cell lines, however it showed no effect when SIRT7 was similarly overexpressed in p53 knockout Huh7.5 cells or p53-null Hep3B cells. Huh7.5 cell carries point mutation at codon 220 (Y220C) of p53 [29, 47], however, our data indicated that SIRT7-dependent p53 regulation is not affected by this mutation at least in the case of doxorubicin treatment. In clinical samples, we observed that high SIRT7 expression is correlated with poor outcomes after TACE treatment. Most importantly, we found that inhibiting SIRT7 by knockdown or pharmacological inhibitors significantly increased p53 activation and doxorubicin induced cell death. Our data clearly demonstrate the important role of SIRT7 in regulating HCC therapy sensitivity. Therapeutically targeting SIRT7 may thus offer options for enhancing the efficacy of HCC treatment.

It has long been recognized that the function of p53 is regulated by acetylation. Enhancement of p53 acetylation strongly correlates with protein stabilization and activation in response to cellular stress [27]. Multiple SIRT proteins have been reported to deacetylate p53 including SIRT1 [30], SIRT2 [31], SIRT3 [32], SIRT6 [48] and SIRT7 [8]. Here we show that SIRT7 is a p53 deacetylase both in vitro and in vivo and deacetylation by SIRT7 suppresses p53 transactivation activity. We further demonstrated that SIRT7 deacetylates p53 at K320 and K373, which alters p53 transcriptional programs and specifically turns off p53-dependent NOXA expression. Targeted substitutions at serine 320 and 373 were sufficient to abolish NOXA transcription and glutamine substitutions that mimic acetylation induced p53 binding and NOXA transcription. Neither mutants showed effects on expression of other p53-dependent genes. These data suggest that acetylation of lysines 320 and 373 are critical determinates of NOXA transcription. Our finding thus provides insight into the mechanisms of blockade of the p53-dependent apoptosis machinery in HCC.

NOXA was initially identified as a p53 target gene which plays critical role in p53-mediated apoptosis in cells exposed to noxious stress by regulating mitochondrial stability [49]. Even though the involvement of NOXA in carcinogenic processes remains elusive, its importance in chemoresistance in cancer is becoming increasingly evident [50–53]. Large numbers of cancer cell lines have been reported to efficiently resist chemotherapy through modification of NOXA stability [53–55]. Remarkably, induction of NOXA can sensitize resistant cancer cells to chemotherapeutics [52, 56, 57]. Comparative genomic hybridization (CGH) and microarray analyses have identified mutations or silencing of NOXA in cancer [58]. In the context of doxorubicin, we observed that SIRT7 regulates p53-induced NOXA transcription through p53 deacetylation. Inhibiting SIRT7 by knockdown or pharmacological inhibitors significantly increased NOXA expression and increased cell death. Even though NOXA is the main target gene responsible for the p53-indueced cell death pathway [49], whether NOXA is primarily responsible for SIRT7 dependent protection from doxorubicin requires further investigation.

## Conclusions

In conclusion, we have demonstrated a crucial role of SIRT7 in suppressing p53-dependent cytotoxicity of HCC. SIRT7 inhibition increased the sensitivity of liver cancer cells to cell death inducing chemotherapeutics such as doxorubicin both in vitro and in vivo. Our findings thus highlight the importance of the SIRT7 in HCC carcinogenesis and identify SIRT7 as a potentially useful target for the development of mechanism-based cancer therapeutic strategies.

## Additional files

Additional file 1: **Table S1.** Primer sequences used for RT-PCR. **Table S2.** Primer sequences used for ChIP experiment. (DOCX 17 kb)

Additional file 2: **Figure S1**. Representative protein levels of SIRT7 in normal, cirrhotic and HCC liver sections. **Figure S2**. (A) Immunofluorescence for SIRT7 in Huh7.5 cells treated with doxorubicin for various time. (B-C) Huh7.5 cells were either untreated (UT) or treated with doxorubicin in the absence or presence of various of inhibitor as indicated, SIRT7 mRNA and protein levels were evaluated by RT-PCR and western blot. (D-E) Huh7.5 cells were transfected with flag-tagged plasmids and SIRT7 level were evaluated by immunofluorescence (D) and protein-protein interactions were evaluated by immunoprecipitation (E). Arrowheads indicate transfected cells and arrows indicate untransfected cells. **Figure S3**. (A) Intercellular localization of Flag-taggedSIRT7 or SIRT7 H187Y (SIRT7 HY) and HA-tagged p53 in Huh7.5 cells. (B) Huh7.5 cells were transfected with HA-SIRT7 with WT flag tagged p53 or mutants as indicated, p53 proteins were purified by immunoprecipitation and acetylation levels of p53 were evaluated by western blot. (C) Intercellular localization of p53 wild type (WT), K320,373R (2KR), K320,381,382R (3KR-A), K120,320,373R (3KR-B), K372,373,381,382R(4KR), K120,372,373,381,382R(5R). (D)p53 knockdown Huh7.5 cells were transfected with WT, 2KR, 2KQ(K320,373Q) or 5KR for 24 hours and treated with doxorubicin. p53 levels were evaluated by western blot (upper) and cell death were evaluated by TUNEL assay (lower). **p < 0.01 vs WT/DOX, student’s t-test. **Figure S4**. RGFP966 inhibits SIRT7 activity in vitro. The flag-p53 was purified from cells treated with H_2_O_2_ (400 uM, 1 h). Purified p53 were incubated in the presence or absence of RGFP966(0.5 uM) or in combination with rSIRT7 and NAD^+^. Acetylation and total amount of p53 were assessed by western blot. (PDF 1514 kb)

## Data Availability

All data generated or analyzed during this study are included in this published article [and its additional files].

## Abbreviations

ChIP: Chromatin immunoprecipitation
CHX: Cycloheximide
DOX: Doxorubicin
HCC: Hepatocellular carcinoma
HDAC: Histone deacetylase
IHC: Immunohistochemistry
PHH: Primary human hepatocyte
TACE: Transarterial chemoembolization
TUNEL: Terminal deoxynucleotidyl transferase dUTP nick end labeling
