# Supplementary material for: SIRT7 regulates hepatocellular carcinoma response to therapy by altering the p53-dependent cell death pathway
Source: J Exp Clin Cancer Res. 2019 Jun 13;38:252. doi: 10.1186/s13046-019-1246-4 (PMC6567523; doi:10.1186/s13046-019-1246-4)
Supplement: Supplementary file 1 — Table S1. Primer sequences used for RT-PCR. Table S2. Primer sequences used for ChIP experiment. (DOCX 17 kb) [file 13046_2019_1246_MOESM1_ESM.docx]

**Additional file 1**

***Table S1.* Primer sequences used for RT-PCR.**

| *NOXA forward* | *5'- GTGCCCTTGGAAACGGAAGA-3'* |
| --- | --- |
| *NOXA reserse* | *5'*- *CCAGCCGCCCAGTCTAATCA-3'* |
| *p21 forward* | *5'- CTGGAGACTCTCAGGGCGAAA-3'* |
| *p21 reverse* | *5'- GATTAGGGCTTCCTCTTGGAGAA-3'* |
| *GADD45 forward* | *5'- TGCTCAGCAAAGCCCTGAGT-3'* |
| *GADD45 reverse* | *5'- GCTTGGCCGCTTCGTACA -3'* |
| *BAX forward* | *5'- CCCGAGAGGTCTTTTTCCGAG -3’* |
| *BAX reverse* | *5'- CCAGCCCATGATGGTTCTGAT -3’* |
| *PUMA forward* | *5'- GGGCCCAGACTGTGAATCCT-3’* |
| *PUMA reverse* | *5'- ACTTGCTCTCTCTAAACCTATGCA-3’* |
| *SIRT1 forward* | *5'-TAGCCTTGTCAGATAAGGAAGGA-3'* |
| *SIRT1 reverse* | *5'-ACAGCTTCACAGTCAACTTTGT-3'* |
| *SIRT2 forward* | *5'-TGCGGAACTTATTCTCCCAGA-3'* |
| *SIRT2 reverse* | *5'-GAGAGCGAAAGTCGGGGAT-3'* |
| *SIRT3 forward* | *5'-ACCCAGTGGCATTCCAGAC-3'* |
| *SIRT3 reverse* | *5'-GGCTTGGGGTTGTGAAAGAAG-3'* |
| *SIRT4 forward* | *5'-GCTTTGCGTTGACTTTCAGGT-3'* |
| *SIRT4 reverse* | *5'-CCAATGGAGGCTTTCGAGCA-3'* |
| *SIRT5 forward* | *5'-GCCATAGCCGAGTGTGAGAC-3'* |
| *SIRT5 reverse* | *5'-CAACTCCACAAGAGGTACATCG-3'* |
| *SIRT6 forward* | *5'-CCCACGGAGTCTGGACCAT-3'* |
| *SIRT6 reverse* | *5'-CTCTGCCAGTTTGTCCCTG-3'* |
| *SIRT7 forward* | *5'-GACCTGGTAACGGAGCTGC-3'* |
| *SIRT7 reverse* | *5'-CGACCAAGTATTTGGCGTTCC-3'* |
| *GAPDH forward* | *5'-GAAGGTGAAGGTCGGAGTC-3'* |
| *GAPDH reverse* | *5'-GAAGATGGTGATGGGATTTC-3'* |

***Table S2.* Primer sequences used for ChIP experiment.**

| *NOXA forward* | *5'- CAGCGTTTGCAGATGGTCAA -3'* |
| --- | --- |
| *NOXA reserse* | *5'- CCCCGAAATTACTTCCTTACAAAA -3'* |
| *p21 forward* | *5'- GTGGCTCTGATTGGCTTTCTG -3'* |
| *p21 reverse* | *5'- CTGAAAACAGGCAGCCCAAG -3'* |
| *GADD45 forward* | *5'- AGCGGAAGAGATCCCTGTGA -3'* |
| *GADD45 reverse* | *5'- CGGGAGGCAGGCAGATG -3'* |
| *BAX forward* | *5'- TAATCCCAGCGCTTTGGAA -3’* |
| *BAX reverse* | *5'- TGCAGAGACCTGGATCTAGCAA -3’* |
| *PUMA forward* | *5'- GCGAGACTGTGGCCTTGTGT -3’* |
| *PUMA reverse* | *5'- CGTTCCAGGGTCCACAAAGT -3’* |
