# Supplementary figures and images for: SIRT7 regulates hepatocellular carcinoma response to therapy by altering the p53-dependent cell death pathway
Source: J Exp Clin Cancer Res. 2019 Jun 13;38:252. doi: 10.1186/s13046-019-1246-4 (PMC6567523; doi:10.1186/s13046-019-1246-4)

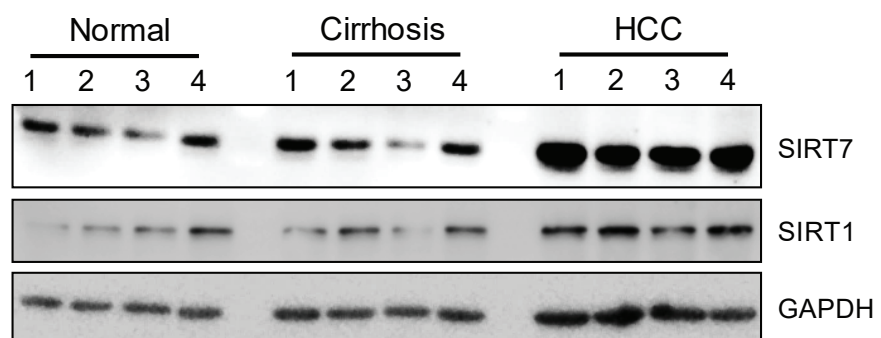

**Zhao et al. Figure S1**

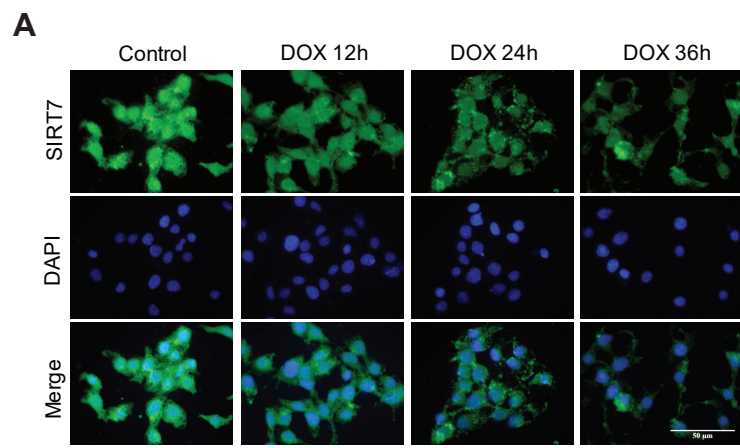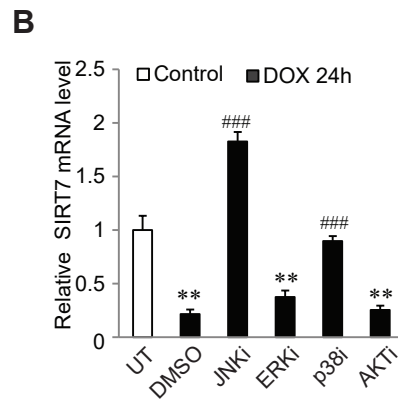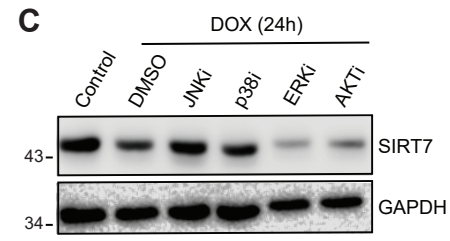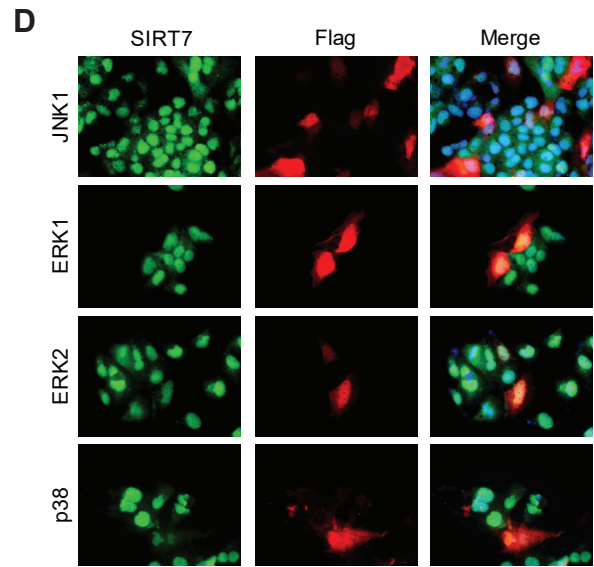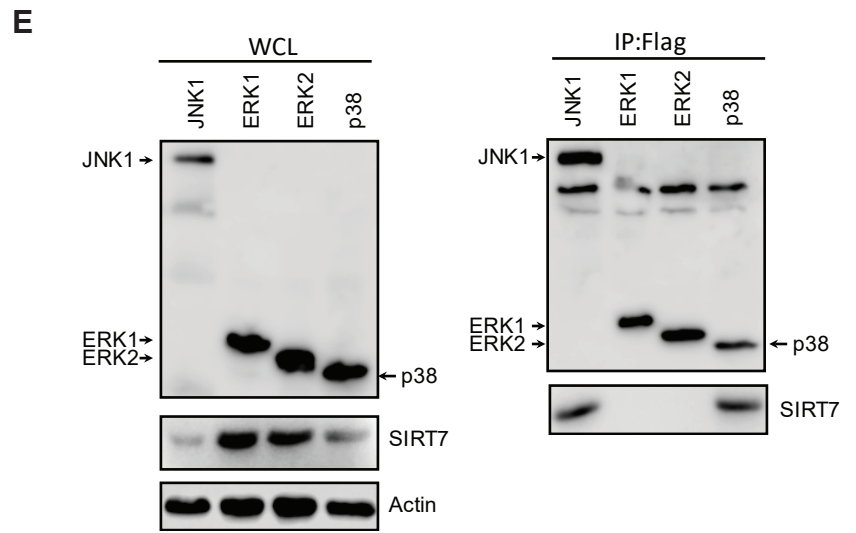

**Zhao et al. Figure S2**

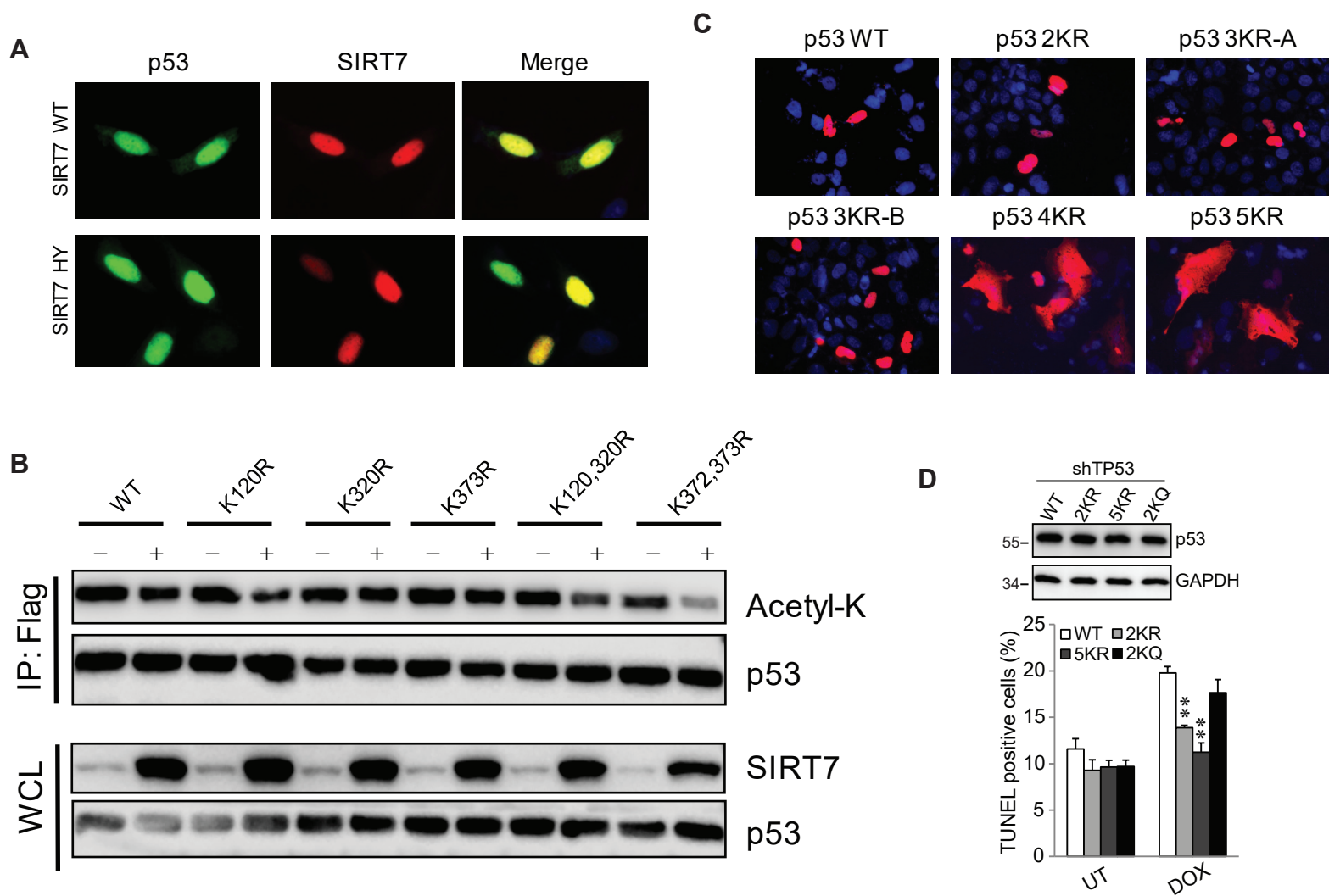

**Zhao et al. Figure S3**

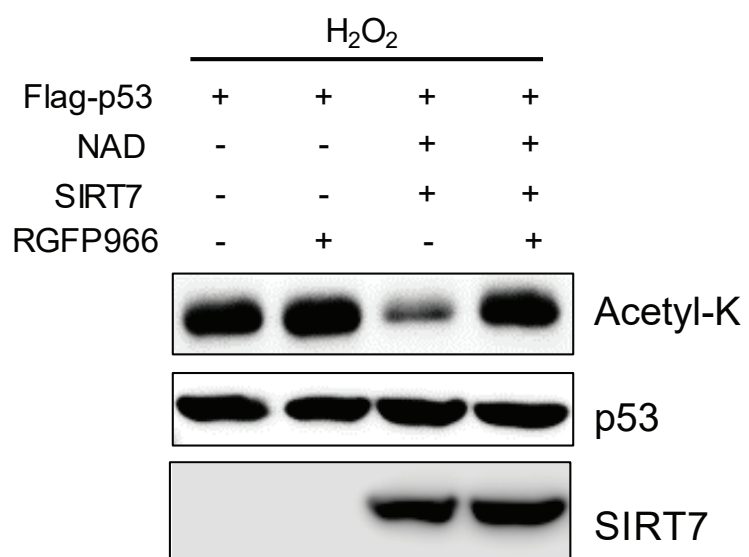

***Zhao et al. Figure S4***

Supplement: Supplementary file 2 — Figure S1. Representative protein levels of SIRT7 in normal, cirrhotic and HCC liver sections. Figure S2. (A) Immunofluorescence for SIRT7 in Huh7.5 cells treated with doxorubicin for various time. (B-C) Huh7.5 cells were either untreated (UT) or treated with doxorubicin in the absence or presence of various of inhibitor as indicated, SIRT7 mRNA and protein levels were evaluated by RT-PCR and western blot. (D-E) Huh7.5 cells were transfected with flag-tagged plasmids and SIRT7 level were evaluated by immunofluorescence (D) and protein-protein interactions were evaluated by immunoprecipitation (E). Arrowheads indicate transfected cells and arrows indicate untransfected cells. Figure S3. (A) Intercellular localization of Flag-taggedSIRT7 or SIRT7 H187Y (SIRT7 HY) and HA-tagged p53 in Huh7.5 cells. (B) Huh7.5 cells were transfected with HA-SIRT7 with WT flag tagged p53 or mutants as indicated, p53 proteins were purified by immunoprecipitation and acetylation levels of p53 were evaluated by western blot. (C) Intercellular localization of p53 wild type (WT), K320,373R (2KR), K320,381,382R (3KR-A), K120,320,373R (3KR-B), K372,373,381,382R(4KR), K120,372,373,381,382R(5R). (D)p53 knockdown Huh7.5 cells were transfected with WT, 2KR, 2KQ(K320,373Q) or 5KR for 24 hours and treated with doxorubicin. p53 levels were evaluated by western blot (upper) and cell death were evaluated by TUNEL assay (lower). **p < 0.01 vs WT/DOX, student’s t-test. Figure S4. RGFP966 inhibits SIRT7 activity in vitro. The flag-p53 was purified from cells treated with H2O2 (400 uM, 1 h). Purified p53 were incubated in the presence or absence of RGFP966(0.5 uM) or in combination with rSIRT7 and NAD+. Acetylation and total amount of p53 were assessed by western blot. (PDF 1514 kb) [file 13046_2019_1246_MOESM2_ESM.pdf]
